# On Asthma—Paralysis—Dropsy

**Published:** 1840-02-22

**Authors:** W. W. Gerhard


### CLINICAL LECTURE.

### PHILADELPHIA HOSPITAL.

*Wednesday, February 12th, 1840.*

### ON ASTHMA—PARALYSIS—DROPSY.

By W. W. GERHARD, M. D.

*No. 13—Winter Course.*

I SHALL speak to-day of certain disorders, which are rather to be considered as the effects of disease, than as diseases in themselves. These disorders are mostly of an obscure and chronic character, and, therefore, not so interesting to the commencing student, as many of those cases of acute and well marked disease, which have heretofore occupied so large a share of our attention. These affections, nevertheless, are of such frequent occurrence in the practice of every physician, that it is of the highest importance that you should be made acquainted with them. Apart from this practical importance, the very obscurity of the lesions, upon which they in many cases depend, should render them objects of interest to every one who studies medicine as a science.

Though it may seem paradoxical at first sight, there is, really, in many instances, great difficulty in discriminating between symptoms and diseases. In many practical works on medicine, various affections are described as diseases, which are, in fact, only symptoms,—that is, manifestations of an organic lesion in some viscus or other of the economy. The most important of these affections, and those to which I wish particularly to call your attention at present, are Asthma, Paralysis and Dropsy. In most works, excepting those very recently published, these are considered as idiopathic diseases. The fact is that those instances are very rare, in which they are truly idiopathic; in the great majority of cases they may be referred to organic lesions of some important internal organ, and there can hardly be a doubt, that further investigations will enable us thus to refer many of those cases which are still considered idiopathic. At the last lecture, I illustrated this matter, so far as it relates to dropsy, somewhat in detail. Dropsy, until very lately, was spoken of as a distinct disease; it is now known never to be so, except in a few rare instances. These may be referred to two classes; 1st, those which arise in consequence of a sudden exposure to cold, and an arrest of the cutaneous exhalation; and 2dly, those which result from inflammation of a serous membrane, however excited; but this last class can scarcely be included among dropsies proper. All other varieties of dropsy, except those just mentioned, are now referred to lesions of the heart or venous system, liver or kidneys; several cases, in illustration of this, were presented to you at the last lecture, and I need not note more fully into the subject at present.

Asthma is now considered as idiopathic, only in those cases which depend apparently upon some functional disorder of the nervous system, and in which no organic lesion can be discovered after death, cf such a kind or degree as to account for the intense dyspnœa which characterizes the disease. Now we have had but a single case during the present season which can be strictly referred to this head. It was that of a man who was subject to violent paroxysms of dyspnœa, together with symptoms of slight inflammation of the bronchial tubes. It is this variety of the disease, which, in New England, is called "rose asthma," and in England, "hay asthma," or "hay catarrh." Such instances as this, are really idiopathic; for in them, the dyspnœa commences as a true nervous affection, and organic affections, if they occur at all, are secondary.

But asthma, in the great majority of cases, is only a symptomatic affection, and depends on an organic lesion of one of the thoracic viscera. When it is a permanent, not a paroxysmal disorder, it arises most frequenty, perhaps, from *emphysema of the lungs*, a lesion of which you have yet seen few or no examples. This variety of asthma is never dangerous, unless it be complicated with phthisis, or some other serious disease of the lungs or heart. When uncomplicated, it is characterized by shortness of breath in ascending a flight of stairs, or in making any other unusual exertion; by the same fatigue and dyspnœa after talking, when the oppression is most obvious at the end of every sentence; and by the physical signs of extreme resonance on percussion, and feebleness of the respiratory murmur. When it exists, however, only in a slight degree, emphysema may pass almost unnoticed for a great length of time.

The anatomical lesion in this disease, consists in an enlargement of the vesicles of the lungs. Of course, if one set of vesicles is greatly enlarged, another set must be atrophied in a corresponding degree. The effect of this dilatation of the vesicles must be, to cause the air to remain longer in its passage in and out of the lung than it ought to. From this delay in the passage of the air, arises the difficulty of breathing. This change in the pulmonary vesicles almost always takes place first and in the highest degree at the anterior margin of the lungs. The remote causes of emphysema are of various kinds. It is sometimes congenital; but in most cases it depends upon some acute disease. Thus pneumonia, which obstructs the action of the central and posterior parts of the lung, necessarily causes an increased expansion of the cells of the anterior part, because these have a double duty to perform: and this temporary expansion frequently becomes permanent, and is aggravated by every cause giving rise to some degree of dyspnœa, or to excessive action of the respiratory organs. Emphysema may likewise be caused by more chronic diseases, such as bronchitis, phthisis, or any other, in fact, which puts the air cells on the stretch for any length of time: these cells having but little resiliency in their parietes, are apt to yield to the expansive force, and suffer a permanent change of capacity.

Again: asthma may be caused by disease of the *heart*. It is difficult to say whether this or emphysema of the lungs is the more frequent cause, because they so commonly coexist in the same individual. Indeed, one of them hardly ever occurs without inducing the other sooner or later. Thus emphysema causes dyspnœa; the blood consequently accumulates about the heart; a disordered action of this viscus is the result, and this again produces in course of time a corresponding change of structure. Or the order may be reversed: the disease may originate in the heart, and be transmitted to the lungs. In either case, the asthma is not an idiopathic disease, but a secondary one; or, more properly, a symptom of an organic lesion of the heart or lungs.

Again: *chronic bronchitis* may give rise to asthma.

The bronchitis in such cases, is of the dry kind, unattended with secretion, but marked by a thickening of the mucous membrane. This opposes a mechanical obstruction to the exit of the air from the lungs, and the vesicles are therefore forced to dilate themselves: in other words, emphysema is the result. This again, as already explained, may in the end cause a disease of the heart, which will tend to aggravate the asthma already produced. Thus we find, that asthma may depend upon various lesions, either as an immediate or a remote consequence. It is a subject of interest to the pathologist, inasmuch as it illustrates the connexion between symptoms and lesions, even when the latter are most remote and obscure.

Paralysis is in a few cases idiopathic, but for the most part it is dependent on an evident lesion of some portion of the nervous system. One of its most frequent causes is *apoplexy*. The paralysis following apoplectic effusion is always of the *hemiplegic* kind,—the paralysis affecting the side opposite to that in which the effusion has taken place. Apoplectic effusions, for the most part, occur in the substance of the corpus striatum or the optic thalamus, but, if sufficiently abundant, they may break into, and even fill the adjacent ventricle. There appears to be no connexion between the particular part of the brain in which the lesion occurs, and the part of the body which is subsequently affected with palsy. The latter generally affects the whole of one side; but the loss of power is perceptible first, and to the greatest degree in the upper extremity; the lower extremity, as it suffers least, so it is the first to recover its normal condition. The paralysis following apoplexy has one pathognomonic character, by which it may be certainly distinguished from all other varieties of the disease; that is, the *suddenness* with which it makes its appearance. Other cerebral lesions occasion paralysis, but never with the perfect suddenness which characterizes this form. The reason is obvious; the sanguineous effusion produces an instantaneous destruction of a portion of the cerebral mass; at the same instant, consequently, there results a loss of function. Hence, in investigating the source of an attack of palsy, we should always inquire first into its mode of origin. If the patient tells that he was suddenly taken with ringing of the ears, giddiness, and loss of consciousness, and immediately afterwards with paralysis, we may conclude, with perfect certainty, that an apoplectic effusion has been the exciting cause of the affection. Its remote cause may be any of those various circumstances which produce a tendency to extravasations of blood from the cerebral vessels; such as intense and long continued exercise of the mind, local injuries, ossification of the arteries of the brain, &c.

*Congestion* of the brain, merely, may also produce paralysis; but in this case it is only temporary, and may generally be cured by efficient depletion. But we can never expect to cure a case of true apoplectic palsy, because it is dependant on the mechanical destruction of a portion of the cerebral substance, which can never be replaced.

Inflammation of the brain, terminating in softening, is another cause of paralysis. As in the case of apoplectic effusion, this affection of one side of the brain, causes paralysis of the opposite side of the body. There are two marks which distinguish these two forms of palsy from each other; that arising from ramollissement, is characterized by rigidity from its very commencement; it also begins slowly and by degress. On the contrary, that following apoplexy is, at first, characterized by relaxation, instead of rigidity, and commences suddenly.

Inflammation of the *membranes* of the brain, involving the cortical substance, may, in like manner, give rise to paralysis, which, as in the preceding case, is marked by rigidity, which lasts as long as the inflammatory affection on which it depends. In old people, again, we have a kind of paralysis, which is produced by softening of some portion of the cerebral tissue; but this disorganization does not depend on a proper inflammation; it is a decay, a sort of *necrosis* of the brain. It commences by degrees; is more permanent than either of the varieties just spoken of; and generally affects both sides of the body at the same time, but in an unequal degree. Its usual cause is ossification, or, at least, cartilaginous transformation of the arteries of the brain.

We have still another form of paralysis, occurring in the *insane*. But it is by no means confined to this class of persons; it is often observed in individuals whose intellect is sound, or at any rate, not sufficiently disordered to entitle it to the appellation of insanity. The insanity, when it occurs, is only one symptom of the lesion giving rise to the paralysis, and may be present or absent, according to circumstances. There are, consequently, two varieties of the complaint. In one, there is at first no insanity, but simply an enfeebled state of the sensibility and power of locomotion, in some part of the body. We sometimes meet with this kind of palsy in men of brilliant intellect, who have accustomed themselves to intense mental effort. It generally comes on gradually, and after progressing to a certain point, it may abort, or it may continue until it effects an entire destruction of the powers of intellect and of locomotion. The loss of power is first manifested in the voice: there is a peculiar hoarseness or huskiness observed at the end of each word or syllable. This change of voice is so peculiar and well marked, as to have received the name of the paralytic tone. This may be the only symptom of the affection for a considerable period. But sooner or later some change in the activity of the intellect is observed, and it is first perceived in most cases, by the patient himself, especially if the memory fails before the other powers. It may amount to positive insanity, or merely to a conscious imbecility of mind. In the latter case, the patient generally takes great pains to conceal it as far as possible, from those around him. In the further progress of the affection, the powers of locomotion become impaired, particularly in the lower extremities, because any weakness in them will manifest itself in walking, in spite of the efforts of the patient: but there not being the same necessity for steadiness in the motions of the arms, any irregularity in these motions may pass unnoticed for some time. The palsy generally affects both sides, and produces a peculiar waddling motion, and straddling of the legs, when the patient walks. The aberration of mind in such cases is often peculiar: the patient is constantly indulging in lofty notions of grandeur, wealth, &c. In another variety of this palsy, the disorder of intellect is first to manifest itself: the patient becomes insane, before he becomes paralytic. The anatomical changes observed in persons who die of this sort of paralysis, consist of thickening and opacity of the membranes, and induration of the cortical substance,—the consequences of chronic inflammation. In the commencement, this palsy is generally curable: but when once fully established, we can entertain no hopes of a favourable result.

Paralysis may likewise be the consequence of an altered nutrition of the substance of the brain, producing scirrhous or tuberculous deposites; or of exostosis, osteo-sarcoma, or other bony tumours of the cranium. There are of course no certain means of distinguishing paralysis arising from these causes. The existence of scirrhus or tubercles in the brain may be suspected, from their simultaneous existence in other parts of the system: but it is impossible to detect exostosis and other lesions of this nature, unless they should happen to present themselves externally. The paralysis arising from these causes resembles in many respects that which is consequent upon the softening of the brain in old people.

All these varieties of cerebral paralysis are often obscure at their onset, and for a time partially latent. You hear sometimes of entire destruction of considerable portions of the cerebral mass, without the occurrence of any symptoms to point it out. These occurred to me from time to time, but of late years, I have scarcely met with them, and am obliged to infer, that I now notice symptoms which would escape an observation not cultivated by long practice.

*Paraplegia*, or paralysis of the lower extremities, arises from some affection of the spinal marrow,—generally inflammation and its consequences, or caries of the vertebræ. It is frequently curable in the first stages by local antiphlogistic and revulsive measures: but if disorganization of the spinal marrow has taken place, the disease may be considered incurable.

The foregoing remarks will show you the various lesions upon which different forms of paralysis depend. Nevertheless there are cases, in which no organic lesion can be discovered. Such are those cases in which the disease attacks not the centre, but the periphery of the nervous system. Thus a single nerve may be alone affected; a finger or toe may be seized with weakness and numbness. These cases of paralysis, as they cannot be accounted for by any known and appreciable lesion, must be considered idiopathic. But it is not improbable, that they may hereafter be referred to lesions as yet undiscovered. These instances are often met with in persons who have injured their intellect by excessive study, or by intemperance. The paralysis following the poison of lead may also be referred to the same variety.

There is another class of cases in which no particular lesion of the nervous system can be detected. Yet they are not idiopathic, but sympathetic of an affection of some thoracic or abdominal organ: they therefore continue only so long as their local cause does. Thus disorders of the stomach, heart, or uterus, may give rise to a temporary paralysis. Cases of this kind are rare in men, but much more frequent in women, and are generally connected with uterine irritation.

The account which I have now given you, of a set of diseases which were, until lately, very imperfectly understood, will aid you materially in the study of them. Without having some definite notions of their pathology, it will be impossible for you to understand the variations which are constantly met with in their course, and the treatment appropriate to each particular case. Before concluding, I wish to present to you some cases of paralysis, as instances of several of the forms of the complaint which I have described.

*Case* 1.—You can easily discover from the conversation and motions of this man, the particular forms of the disease with which he is affected. His mind is clear: the motions of his arms are unimpaired: in the lower extremities only is there a loss of power. His gait is tottering, and he is obliged to use a cane to support himself. The loss of sensibility and motion in the lower extremities was first perceived about a year ago: it commenced very gradually: the patient is ignorant of any cause which may have produced it, unless it be habitual intemperance. I may remark that in these cases of paraplegia, we often seek in vain for a probable cause; as they, in many cases, depend on certain venereal excesses, about which the patient is not very apt to speak freely. The right leg is weaker than the left; differences of this kind are frequently observed, but we seldom see a case in which one leg only is affected. The patient suffers more or less pain in the right ankle, and sometimes in the small of the back; he never has cramp in the legs, but frequently feels a twitching, and darting pains; pricking and creeping sensations are also frequent symptoms of paraplegia; but this patient seems not to have experienced them. He has also had emphysema of the lungs, which followed an attack of pneumonia, and was attended with a tubercular deposition. But the progress of the latter formation has been almost arrested since the paralysis commenced. From the fact of the previous existence of a tuberculous affection, we are enabled to account for the occurrence of the paralysis. The connexion between a scrofulous diathesis and caries of the vertebra is well known; and it is probable that such an affection of the spinal column exists in the present instance, attended by inflammation of the spinal marrow itself. Some signs of the former emphysema are still perceptible; the percussion is very resonant on the anterior walls of the chest; the respiratory murmur is feeble; and on the left side there is a decided fulness of the ribs.

*Case* 2.—This is the man whom you saw about two months since, with a prominence of the spinous processes in the lower part of the dorsal region. The treatment has consisted of the application of moxas and blisters, purging, and, what in such cases is more important than any thing else, *rest.* The local pain has been gradually declining. During the course of the disease, the patient has suffered much from neuralgic pains in the lower extremities: in addition to the remedies already mentioned, these pains were, on one occasion, on account of their paroxysmal character, treated with sulphate of quinine, and with complete success. The swelling on the back is now nearly gone, and the cure may be considered as complete. The paralysis in this case, too, depended on inflammation of the spinal marrow consequent on disease of the vertebræ.

*Case* 3.—This woman has been paralytic in the right arm and leg for two or three years. While in the enjoyment of perfect health, she was suddenly attacked one day with giddiness and loss of consciousness. From this attack she dates the beginning of the paralysis. The loss of feeling and strength have much diminished of late: the numbness principally affects the thumb and fingers of the right hand; the right leg is also somewhat weak. Cups were first applied to the back; but, as they seemed to produce little relief, they were next applied high up in the cervical region. This treatment has been attended with considerable improvement. The hemiplegia is, in this case, evidently owing to an apoplectic effusion, which has partially destroyed a portion of the cerebral substance.

*Case* 4.—This old man has been paralytic for two years; the disease commenced gradually. His gait is very tottering; the right arm is weak; the mental faculties much enfeebled, and the paralytic tone of voice very well marked. This is probably a case of the paralysis of the insane, depending on chronic inflammation of the membranes and substance of the brain.

I next show the dropsical patient who was before you at the last lecture. You will recollect that I then stated that the dropsical effusion was caused by a disease of the kidneys, which was indicated by the presence of albumen in the urine. It has been treated principally by cupping over the kindneys. This treatment has produced a very decided reduction of the swelling: the remains of it are more evident in the face than in any other part of the body. But the duration of the disease must still be considered very uncertain.

The last case which I shall present you is one of pneumonia. The patient was yesterday admitted, with slight cough, and pain in the lower part of the right axilla; the dyspnœa was very trifling. The disease began only one day before his entrance, and it was very difficult to say, from an examination of the symptoms merely, whether he had pneumonia, bronchitis, or pleurisy. I therefore resorted to an examination of the physical signs, and this revealed the existence of pneumonia. The respiration is imperfectly bronchial at the lower part of the right lung, with feebleness of the vesicular murmur, and crepitant rhonchus. There is but little excitement of the pulse, and the skin is very little above the natural temperature. The inflammation appears, therefore, to be of an asthenic kind, from its very commencement. It has not yet advanced to the second stage, or that of hepatization; if it had, the respiration would have been more decidedly bronchial, and the percussion deficient in resonance. It will probably be unnecessary to employ active measures in a case like the present. I shall therefore order cups to the chest, and put the patient on the internal use of an infusion of the asclepias tuberosa, or pleurisy root. This article is an excellent diaphoretic in such cases, and acts very much in the same way with the eupatorium. Small doses of tartarized antimony would also be of service. The prognosis of this case is altogether favourable, and the duration will be less than the average.

